# Effects of Laser Radiation on Mitochondria and Mitochondrial Proteins Subjected to Nitric Oxide

**DOI:** 10.3389/fmed.2018.00112

**Published:** 2018-04-23

**Authors:** Anatoly N. Osipov, Tatiana V. Machneva, Evgeny A. Buravlev, Yury A. Vladimirov

**Affiliations:** ^1^NI Pirogov Russian National Research Medical University, Moscow, Russia; ^2^IM Sechenov First Moscow State Medical University, Moscow, Russia; ^3^MV Lomonosov Moscow State University, Moscow, Russia

**Keywords:** nitric oxide, laser therapy, hemoproteins, mitochondria, liver, mitochondrial proteins

## Abstract

The biological roles of heme and nonheme nitrosyl complexes in physiological and pathophysiological conditions as metabolic key players are considered in this study. Two main physiological functions of protein nitrosyl complexes are discussed—(1) a depot and potential source of free nitric oxide (NO) and (2) a controller of crucial metabolic processes. The first function is realized through the photolysis of nitrosyl complexes (of hemoglobin, cytochrome *c*, or mitochondrial iron–sulfur proteins). This reaction produces free NO and subsequent events are due to the NO physiological functions. The second function is implemented by the possibility of NO to bind heme and nonheme proteins and produce corresponding nitrosyl complexes. Enzyme nitrosyl complex formation usually results in the inhibition (or enhancement in the case of guanylate cyclase) of its enzymatic activity. Photolysis of protein nitrosyl complexes, in this case, will restore the original enzymatic activity. Thus, cytochrome *c* acquires peroxidase activity in the presence of anionic phospholipids, and this phenomenon can be assumed as a key step in the programmed cell death. Addition of NO induces the formation of cytochrome *c* nitrosyl complexes, inhibits its peroxidase activity, and hinders apoptotic reactions. In this case, photolysis of cytochrome *c* nitrosyl complexes will reactivate cytochrome *c* peroxidase activity and speed up apoptosis. Control of mitochondrial respiration by NO by formation or photolytic decay of iron–sulfur protein nitrosyl complexes is an effective instrument to modulate mitochondrial metabolism. These questions are under discussion in this study.

## Introduction

The mechanisms of physiological activity of nitric oxide (NO) have been the crucial questions in the NO metabolic pathways in the latest two decades. One of the main questions was how NO, a short-lived free radical could survive in cell and perform its physiological activity? Really, NO can easily interact with oxygen, thiols, metals etc., producing substances that are lacking NO physiological characteristics. It was found that NO can exist in cells in complexes with thiols (nitrosothiols) or heme and nonheme iron compounds (heme and nonheme nitrosyl complexes), etc. (1–4). The physiological role of nitrosyl complexes in mitochondria and cells has been the main goal for researchers for many years (5–7). Today, it becomes clear that mitochondrion nitrosyl complexes can play various roles from NO depot up to enzymatic activity controller. An important question regarding the physiological role of nitrosyl complexes, in this case, could be the issue of nitrosyl complex stability as well as how to release free NO from these species.

One of the possible ways to release NO from nitrosyl complexes is the photolysis by means of low power laser radiation (within 100 mW) or photobiomodulation. The problems of laser radiation effects on nitrosyl and other complexes were intensively studied by various research groups (8–12). Based on the similarity of laser radiation action spectra in cells and tissues and the absorption spectra of cytochrome *c* oxidase, it was concluded that, namely, cytochrome *c* oxidase is the main target of the laser radiation and irradiation of cytochrome *c* oxidase is the basic cause of most biological effects (9, 13). The hypothesis that cytochrome *c* oxidase is the primary target of low power laser irradiation also explains the prevalence of He–Ne lasers (632.8 nm) in clinical practice, as the red light has deeper penetration into the biological tissues, due to lower absorption and scattering, if compared with green or blue laser light.

Lately, one more biological mechanism of laser light effects was demonstrated. It was shown that opsins, a light-sensitive G-proteins, present in cells can control light-gated ion channels and thus transduce laser effects. Activation of transient receptor potential channels causes nonselective plasma membrane permeabilization to calcium, sodium, and magnesium ions (14). Recently, it was shown that well-known family of biological chromophores—cryptochromes can transform laser light into the enzymatic activity directed to DNA repair in bacteria (15).

Another important mechanism of laser effects based on the photosensitized membrane lipid peroxidation exists. Laser radiation can be absorbed by porphyrins (protoporphyrin IX derivatives) present in the membranes. The excited porphyrins can induce lipid peroxidation in the phagocyte plasma membrane lipids, this process will increase the membrane permeability to Ca^2+^ ions and subsequent increase of Ca^2+^ concentration in the cytoplasm. Elevated Ca^2+^ concentration activates enzymes and stimulates the production of active oxygen species by phagocytes. This mechanism was experimentally proven, and it is the basis of laser mediated wound healing (16).

On the other hand, according to our experimental studies, there are two most effective mechanisms of low power laser effects in biological systems—(1) heme and nonheme nitrosyl complex photolysis and (2) photodynamic effects of laser radiation mediated by photosensitizers and lipid peroxidation of the membranes (8, 17). The first mechanism is based on the free NO release upon laser photolysis and the second one—on the porphyrin mediated lipid peroxidation of biological membranes and subsequent activation of phagocytic cells. The main goal of this study is to consider the mechanisms of most important physiological and pathophysiological processes, mediated by nitrosyl complexes of various origin and their photolytic decomposition.

## Hemoprotein Nitrosyl Complex Photolysis as a Source of Free NO

Along with NO synthesis by cellular NO synthases and reduction of nitrates and nitrites, nitrosyl complex decomposition serves as a source of free NO in cells. NO moiety of nitrosyl complexes is bound to iron ion in the reduced state, and iron ion belongs to heme in hemoproteins (hemoglobin or cytochromes) or nonheme proteins (usually nonheme iron–sulfur proteins in electron transport chain centers of mitochondria)

Cyt c(Fe3+) −​NO→ Cyt c(Fe3+) + NO⋅

**Figure 1:**
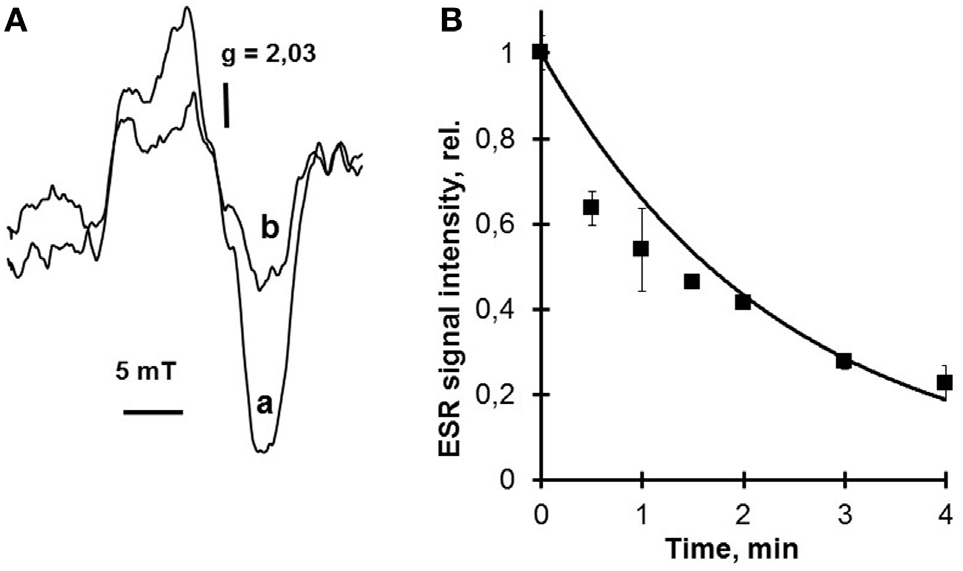
Photolysis of cytochrome *c* nitrosyl complexes. This figure proves the photosensitivity of heme nitrosyl complexes to low power laser radiation. The electron spin resonance (ESR) signals of cytochrome *c* nitrosyl complexes before and after He–Cd laser (442 nm) irradiation in the air are presented in this figure. The ESR signal of cytochrome *c* nitrosyl complex shown in panel **(A)** completely corresponds to hemoprotein nitrosyl complex signal and has *g* factor 2.03 (20). Irradiation of cytochrome *c* nitrosyl complexes by He–Cd laser in the air results in nitrosyl complex photolysis and the corresponding decrease of nitrosyl complex ESR signal **(B)**. Moreover, nitrosyl complex photolysis is accompanied with free nitric oxide (NO) release (data not shown) (18). To detect the NO release, the nitronyl–nitroxyl spin trap can be used. **(A)** ESR spectra of cytochrome *c* nitrosyl complexes. Effects of low power He–Cd (442 nm) laser irradiation: a—no irradiation; b—30 s irradiation. Cytochrome *c* concentration—0.3 mM. He–Cd laser fluence 230 W/m^2^. **(B)** Effect of He–Cd laser radiation on the intensity of NO–Cyt *c* ESR signal. Laser fluence—205 W/m^2^. Cytochrome *c* concentration—0.3 mM.

Hemoprotein nitrosyl complex photolysis is a reversible process. Irradiation of these complexes in the absence of oxygen results in a very low free NO output due to the reverse reaction. But if photolysis takes place in the air, the photolyzed NO interacts with oxygen and does not reassociate with heme iron. Hemoprotein nitrosyl complexes are paramagnetic, and it is easy to detect them by electron spin resonance (ESR) assay. This is exemplified by the studies using hemoglobin and particularly cytochrome *c* nitrosyl complexes, which were accomplished in our laboratory (18), (Figure 1).

Interaction of the nitronyl–nitroxyl spin trap with NO converts it into the imino-nitroxyl compound and changes 5-line ESR signal into the 7-line ESR signal. These results prove our hypothesis that hemoprotein nitrosyl complexes and among them cytochrome *c* nitrosyl complexes are decomposed during photolysis with free NO release and thus they can serve as a NO depot. It is significant that the amount of heme nitrosyl complexes decayed was proportional to the amount of free NO released and trapped by the nitronyl–nitroxyl spin trap. This fact means that mainly heme nitrosyl complexes and not *S*-nitrosothiols undergo decomposition.

## Cytochrome *c*-Cardiolipin (CL) Nitrosyl Complex Photolysis as an Instrument to Control the Cytochrome *c* Peroxidase Activity

**Figure 2:**
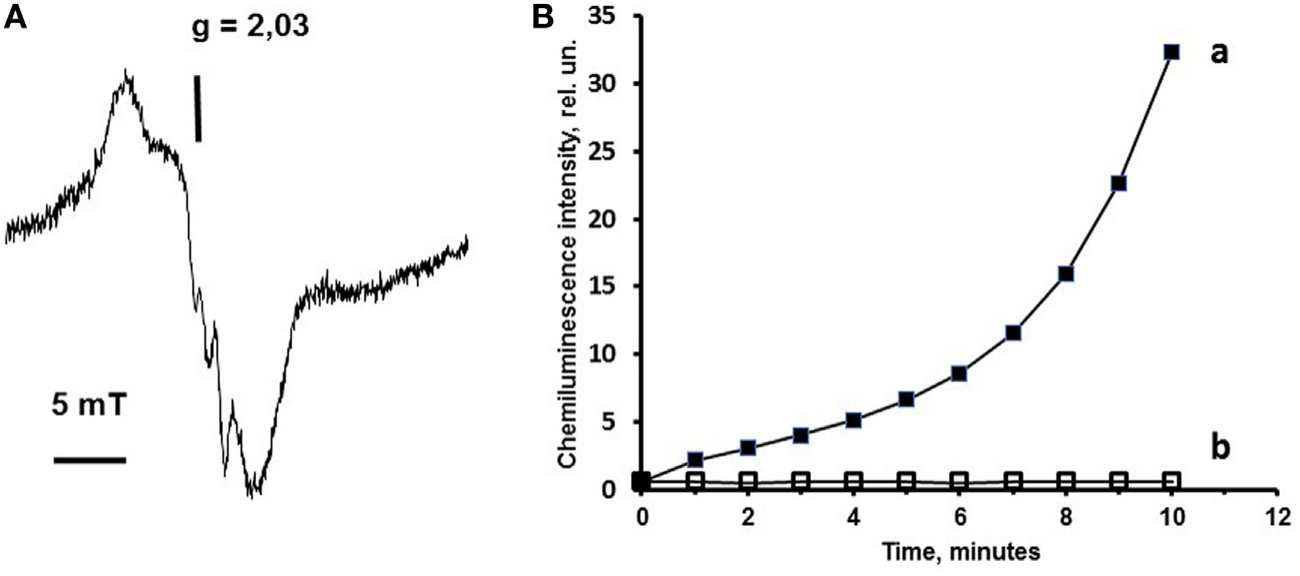
Effect of nitric oxide (NO) on cytochrome *c* properties. The cytochrome *c* nitrosyl complex formation and inhibition of cytochrome *c* peroxidase activity by NO is exemplified by the findings previously published elsewhere (20, 21). In panel **(A)**, a cytochrome *c*–cardiolipin (CL) nitrosyl complex electron spin resonance (ESR) signal is presented. This signal of cytochrome *c* nitrosyl complexes has a *g* factor 2.03 and completely corresponds to nitrosyl complex ESR signal of some hemoproteins. Chemiluminescence assay used in our studies allows to measure the peroxidase activity of cytochrome *c* in the presence of hydrogen peroxide. In panel **(B)**, a chemiluminescent kinetic curve is presented to demonstrate the peroxidase activity of cytochrome *c*–CL complex. **(A)** ESR spectrum of cytochrome *c* nitrosyl complexes formed in the presence of CL. Sample content: cytochrome *c* (50 µM), CL–phosphatidylcholine liposomes (1:1, 0.5 mM), and NO solution in phosphate buffer (0.2 mM). Sample volume 0.5 ml. ESR spectra were recorded at 77 K. **(B)** Effects of CL on cytochrome *c* peroxidase activity measured by chemiluminescence. Sample content: (a) cytochrome *c* (10 µM) + CL–phosphatidylcholine liposomes (1:1, 500 µM); (b) cytochrome *c* (10 μM) + CL–phosphatidylcholin liposomes (1:1, 500 µM) + NO (0.7 mM). All samples were prepared in 10 mM phosphate buffer (pH 7.4). H_2_O_2_ (100 μM) and luminol (500 µM) were added immediately before measurements.

One more crucial physiological process in mitochondria-mediated programmed cell death is the regulation of cytochrome *c* peroxidase activity by means of NO. The peroxidase activity of cytochrome *c* is the vital characteristic of cytochrome *c* in the process of the apoptotic reaction development. It is well known that the interaction of cytochrome *c* with negatively charged phospholipids (such as CL, phosphatidylserine, phosphatidic acid, and some others) leads to the dramatic rearrangement of the cytochrome *c* active site and the acquisition of peroxidase activity, i.e., the ability of cytochrome *c* to oxidize substrates in the presence of hydrogen peroxide (19). In details, this event is based on the phenomenon that interaction of negatively charged CL (−2) with positively charged cytochrome *c* (+8) results in the displacement of methionine-80 in the active site and making vacant the sixth ligand position of heme iron in the active site. This ligand position could be occupied by hydrogen peroxide for peroxidation reaction or NO. NO, in contrast to hydrogen peroxide, forms a strong bond with heme iron, and in this case, the cytochrome *c* peroxidase activity is inhibited. The NO–heme iron complex is, in fact, the nitrosyl heme iron complex, Figure 2A. To destroy this nitrosyl complex and remove it from the cytochrome *c* active site, a low power laser radiation can be applied.

Luminol (chemiluminescence enhancer) present in the sample served as the substrate of peroxidase reaction and produced light upon oxidation. In Figure 2B, the chemiluminescence (line a) increases that corresponds to the increase of the cytochrome *c* complex peroxidase activity. Addition of NO to the sample (line b) inhibits the peroxidase activity of cytochrome *c*–CL complex due to the production of nitrosyl cytochrome *c* (Figure 2B).

**Figure 3:**
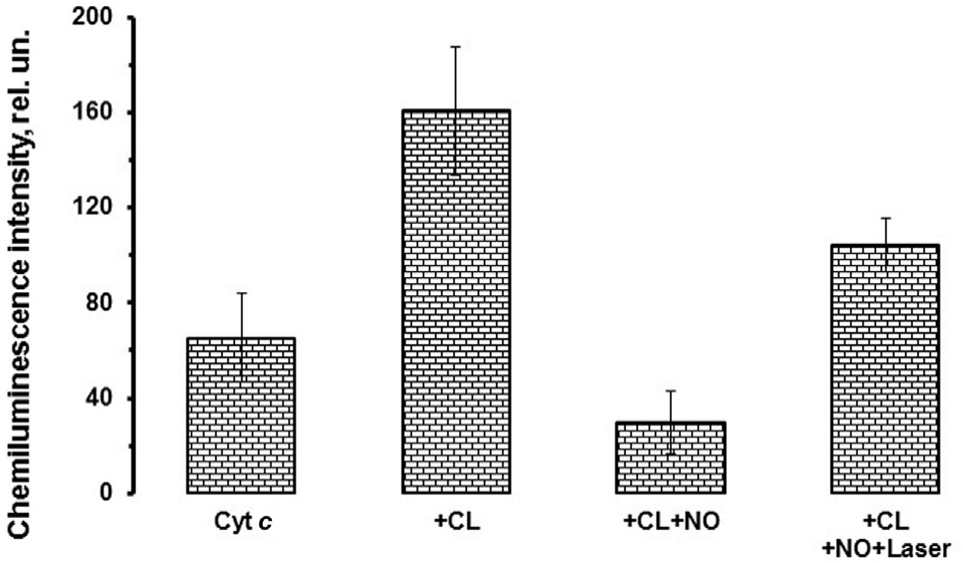
Effects of nitric oxide (NO) and laser radiation on cytochrome *c* peroxidase activity. The cytochrome *c*–cardiolipin (CL) nitrosyl complexes as well as cytochrome *c* nitrosyl complexes produced in the absence of CL possess photosensitivity and can be photolyzed upon irradiation with low power laser radiation. This figure shows the histogram depicting the cytochrome *c* peroxidase activity in the presence or absence of NO and laser radiation. Sample content: cytochrome *c* (10 μM) (most left column, marked as “Cyt *c*”); same as previous sample, but CL–phosphatidylcholine liposomes (1:1, 0.2 mM) were added (second from the left column, marked as “+CL”); same as previous sample, but NO (0.1 mM) was added (third from the left column, marked as “+CL + NO”); same as previous sample, but He–Cd laser (442 nm) irradiation (20 mW, 15 min) was applied (most right column, marked as “+CL + NO + Laser”). All samples were prepared in 10 mM phosphate buffer (pH 7.4). Incubation time was 10 min. H_2_O_2_ (100 µM) and luminol (500 µM) were added immediately before measurements.

Figure 3 shows the integral chemiluminescence of cytochrome *c*–CL complex and NO and He–Cd laser effects on the cytochrome *c* activity. Possible to see that addition of hydrogen peroxide to the cytochrome *c* complex induces the development of cytochrome *c* peroxidase activity, and the addition of NO inhibits the peroxidase activity of cytochrome *c*. Consequently, laser irradiation of the nitrosylated cytochrome *c*–CL nitrosyl complexes destroys nitrosyl complexes and partly restores the cytochrome *c* peroxidase activity (Figure 3).

Analysis of these results allows concluding that hemoprotein nitrosyl complex photolysis can serve not only as an instrument to produce free NO but also to control the enzymatic activity of mitochondrial proteins in the presence of NO. Thus, we can suggest that NO can modulate not only peroxidase activity of cytochrome *c* but also programmed cell death reactions.

## Mitochondria Nitrosyl Complex Photolysis is the Technique to Control Mitochondria Respiration

**Figure 4:**
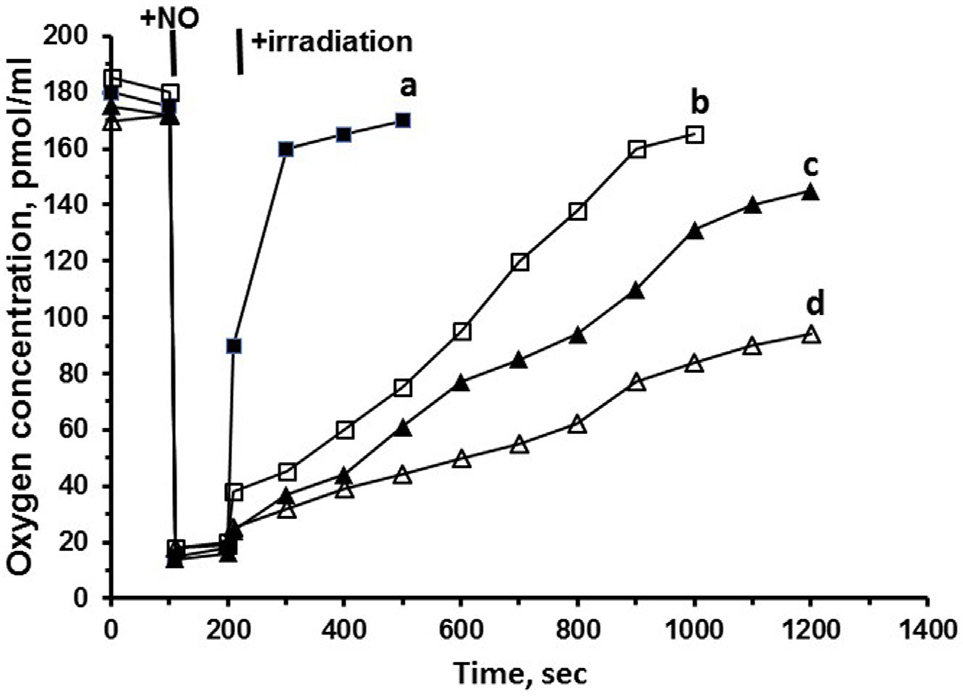
Effects of nitric oxide (NO) and laser irradiation on mitochondrial respiration. The studies of the regulatory physiological mechanisms of NO in mitochondria show that binding of NO by mitochondrial proteins results in the production of photosensitive nitrosyl complexes. The application of laser light irradiation is an effective approach to nitrosyl complex decay. The efficacy of laser photolysis depends on the wavelength of the radiation applied. This issue was addressed in a previous study performed in our laboratory (22). Rat liver mitochondria [0.1 mg of protein/ml in a buffer consisting of 105 mM KCl, 20 mM Tris–HCl, 1 mM diethylenetriaminepentaacetic acid, 5 mM KH_2_PO_4_, and 1 mg/ml fatty acid-free bovine serum albumin (pH 7.4)] were incubated in a temperature-controlled respirometer Oxygraph-2k and illuminated with blue (442 nm) (a) and green (532 nm) (b) lasers or red (650 nm) (c) LED light, or were not illuminated (d). Where indicated 25 µM of NO in physiologic solution was added. Control samples were prepared without laser irradiation.

Figure 4 shows the results of experiments on oxygen consumption by mitochondria, subjected to NO and subsequent low power laser irradiation. Irradiation of mitochondria (subjected to NO) with blue (442 nm) and green (532 nm) lasers or red (650 nm) LED light induces pronounced reactivation of oxygen consumption (Figure 4, curves a–d). The highest effectiveness was demonstrated by blue laser radiation, then by green laser, and finally, by red laser radiation. It is important to mention that the lowest reactivation effectiveness was demonstrated by the red laser irradiation. On the other hand, the red laser has the deepest permeability of light into the tissues that increases the respiration reactivation degree of mitochondria.

Coming to the conclusions based on the experiments on the respiration reactivation ability of laser light, we can state that laser irradiation can serve as an effective instrument to restore the respiration of mitochondria subjected to NO. The results open the possibility of practical application of laser radiation to facilitate the clinical cases with the excessive NO synthesis (endotoxic shock) and increased formation of nitrosyl complexes in mitochondria and other organelles. On the other hand, these results raise the question on the origin of mitochondria nitrosyl complexes: do these complexes originate from hemoproteins, or they have nonheme nature. These questions are discussed in the next section.

## Mitochondria Nitrosyl Complex Photolysis in Experimental Endotoxic Shock

**Figure 5:**
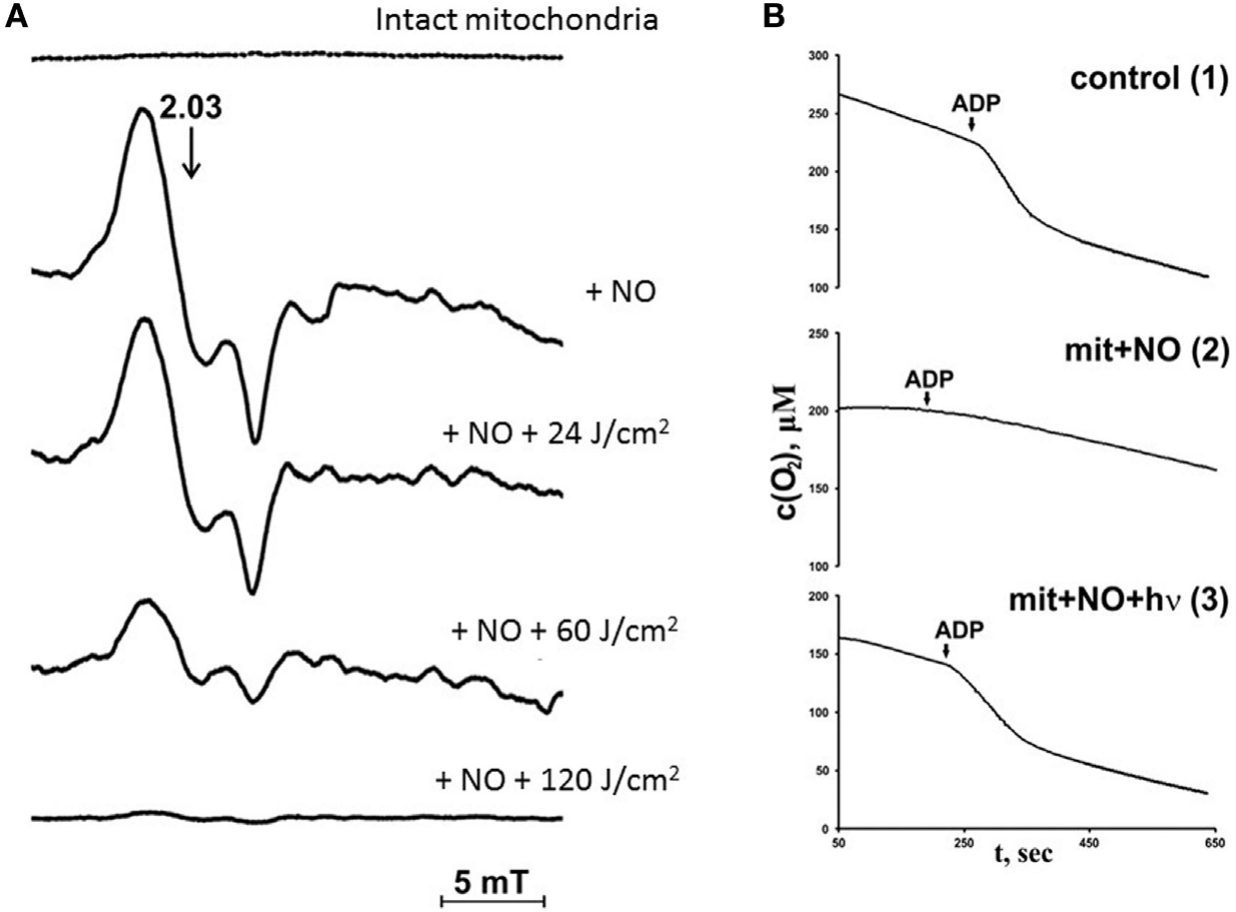
Effects of nitric oxide (NO) and laser radiation on rat liver mitochondria respiration under hypoxic conditions [reproduced with the permission of Springer Publisher from Buravlev et al. (24)]. **(A)** Effects of He–Cd laser (442 nm) irradiation and NO on mitochondria nitrosyl complex electron spin resonance signal. Top line—control mitochondria (1.0 mg protein/ml), next line down mitochondria + NO (3 mM); next lines down—mitochondria + NO + laser irradiation (24, 60, and 120 J/cm^2^, respectively). The hypoxic conditions were produced by incubation of mitochondria during 10 min with an excess of 0.8 mM ADP and 10 mM sodium succinate in storage buffer [the content of the storage buffer is in the legend to panel **(B)**], in air isolated cell. **(B)** Kinetic curves of oxygen uptake in mitochondria (1.0 mg protein/ml). 1—Intact mitochondria (control); 2—mitochondria subjected to 3 mM NO; and 3—mitochondria subjected to 3 mM NO and laser irradiation (λ = 442 nm, 30 J/cm^2^). Incubation medium contained 0.2 µM ADP and 10 mM succinate in the storage buffer (pH 7.45, 10 mM HEPES, 250 µM sucrose, 1 mM ATP, and 2 mM K_2_HPO_4_).

In previous studies, the nature of nitrosyl complexes formed in mitochondria upon interaction with NO were analyzed using ESR assay (23, 24). It was found that mitochondria incubation with NO induces the occurrence of ESR signal with *g* factor 2.03, but different of that produced by cytochrome *c* (Figure 5A). This ESR signal corresponds to the nitrosyl complexes of nonheme iron–sulfur proteins in mitochondria (25). In parallel with nitrosyl complex increase due to the interaction of mitochondrial proteins with NO, the mitochondria respiration rate decreased (Figure 5B). This fact proves that NO interacts with nonheme mitochondria proteins and can inhibit mitochondria respiration.

Nitrosyl complexes detected in mitochondria due to the incubation with NO can be identified as nitrosyl complexes of mitochondrial iron–sulfur proteins. These nitrosyl complexes as well as hemoprotein nitrosyl complexes possess photosensitivity and can be photolyzed by low power laser radiation. The results of the photolytic reaction are displayed in Figures 5A,B. Possible to see that laser radiation destroys mitochondria nitrosyl complexes and restores mitochondria oxygen consumption (Figures 5A,B).

**Figure 6:**
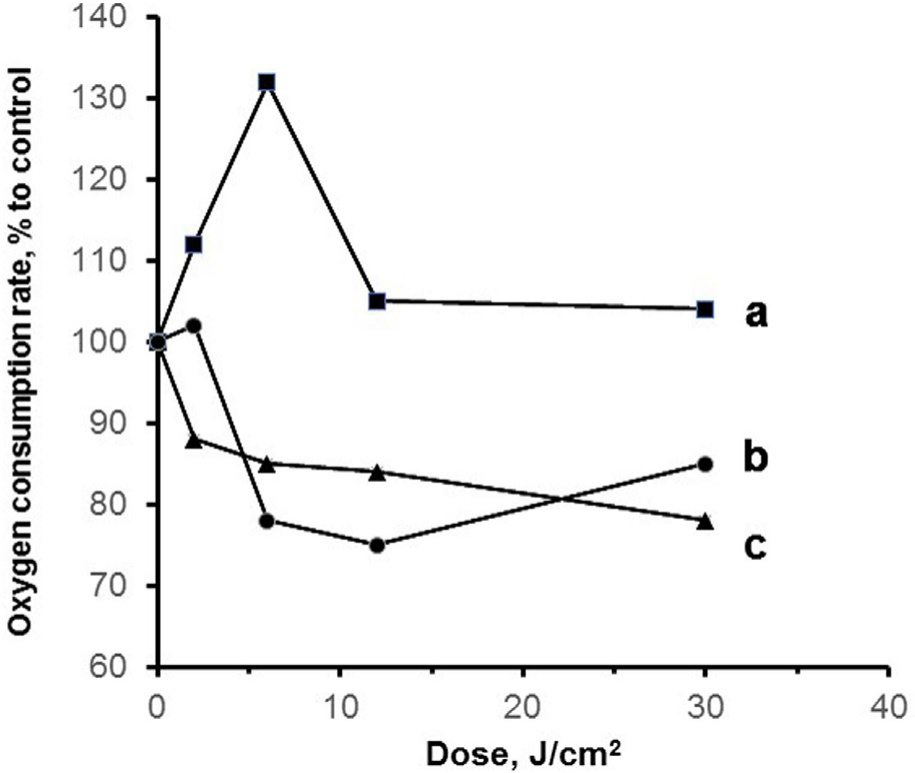
Mitochondria respiration in experimental endotoxic shock [reproduced with the permission of Springer Publisher from Buravlev et al. (26)]. The experimental model of endotoxic shock was produced by intraperitoneal injection of 40 mg/kg lipopolysaccharide B from *Escherichia coli* 0111:B4 (Sigma-Aldrich). Rat liver mitochondria (1.0 mg protein/ml) were isolated and kept on ice in storage buffer (buffer content see in the legend to Figure 4), then immediately used in the experiments. Isolated mitochondria were irradiated with visible light: a—blue 442 nm and b—green 532 nm lasers or c—red 650 nm LED. Dose dependence of relative oxygen consumption rate by mitochondria in state 3 in the presence of ADP (0.2 mM) and succinate (10 mM) as the substrate of oxidation is presented. Control samples were not subjected to laser radiation.

Mitochondrial nitrosyl complexes can be produced not only by exogenous NO addition, but it is also possible to stimulate NO synthesis by intraperitoneal injection of lipopolysaccharide B. This is widely used experimental endotoxic shock model (26). The results of this study are presented in Figure 6. One can see that the induction of experimental endotoxic shock decreased the mitochondria respiration rate and irradiation of mitochondria with blue laser radiation restored mitochondrial respiration. The green (532 nm) laser and red (650 nm) LED were much less effective, and the decreased relative oxygen consumption can be explained by the existence of iron–sulfur protein nitrosyl complexes undestroyed upon laser irradiation.

These results prove that in experimental endotoxic shock model laser radiation can serve as a powerful instrument to improve mitochondrial respiration as it destroys nitrosyl complexes formed in the case of excessive NO production. We can expect that the application of blue lasers (442 nm) in severe intoxications mediated by the formation of nitrosyl complexes in blood or mitochondrial heme or nonheme proteins can prove its higher effectiveness compared with red laser irradiation.

## Conclusion

The previous studies have shown the important physiological role of nitrosyl complexes of hemoproteins and nonheme compounds at least as NO depot (2). Due to the photosensitivity of these complexes, we can expect that low power laser radiation can serve as an effective instrument to locally produce free NO in cells and tissues. It becomes obvious that free NO released from nitrosyl complexes can inhibit the activity of cytochrome *c* (as mitochondrial electron carrier) or mitochondrial iron–sulfur proteins or activate guanylate cyclase. It is important that due to the small physiological concentrations of nitrosyl complexes in cells and tissues low irradiation doses will produce low NO amounts, which will enhance the microcirculation or mitochondrial respiration. But if we will increase the irradiation dose the concentration of NO will be increased too, and finally, we can reach the situation when laser radiation will suppress the microcirculation in blood vessels (3). The mechanism of this phenomenon is the generation of peroxynitrite upon the interaction of free NO excess with superoxide radicals. In our previous publications, we have demonstrated that hemoglobin nitrosyl complexes can be used to improve the microcirculation in tissues (27). Thus, hemoglobin nitrosyl complexes can be used to enhance flap engraftment in skin transplantation or plastic surgery. So, nitrosyl complex photolysis can be a really powerful instrument in some pathological cases mediated by NO.

## Author Contributions

AO, TM, EB, and YV participated in every step of planning and performing the experiments, analyzing the data, writing the manuscript, and preparing the figures.

## Conflict of Interest Statement

The authors declare that the research was conducted in the absence of any commercial or financial relationships that could be construed as a potential conflict of interest.
